# Technology, Privacy, and User Opinions of COVID-19 Mobile Apps for Contact Tracing: Systematic Search and Content Analysis

**DOI:** 10.2196/23467

**Published:** 2021-02-09

**Authors:** Mahmoud Elkhodr, Omar Mubin, Zainab Iftikhar, Maleeha Masood, Belal Alsinglawi, Suleman Shahid, Fady Alnajjar

**Affiliations:** 1 School of Engineering and Technology Central Queensland University Sydney Australia; 2 School of Computer, Data and Mathematical Sciences Western Sydney University Rydalmere Australia; 3 Department of Computer Science Syed Babar Ali School of Science and Engineering Lahore University of Management Sciences Lahore Pakistan; 4 Department of Computer Science and Software Engineering College of Information Technology United Arab Emirates University Alain United Arab Emirates

**Keywords:** contact tracing, COVID-19, digital contact tracing apps

## Abstract

**Background:**

Many countries across the globe have released their own COVID-19 contact tracing apps. This has resulted in the proliferation of several apps that used a variety of technologies. With the absence of a standardized approach used by the authorities, policy makers, and developers, many of these apps were unique. Therefore, they varied by function and the underlying technology used for contact tracing and infection reporting.

**Objective:**

The goal of this study was to analyze most of the COVID-19 contact tracing apps in use today. Beyond investigating the privacy features, design, and implications of these apps, this research examined the underlying technologies used in contact tracing apps. It also attempted to provide some insights into their level of penetration and to gauge their public reception. This research also investigated the data collection, reporting, retention, and destruction procedures used by each of the apps under review.

**Methods:**

This research study evaluated 13 apps corresponding to 10 countries based on the underlying technology used. The inclusion criteria ensured that most COVID-19-declared epicenters (ie, countries) were included in the sample, such as Italy. The evaluated apps also included countries that did relatively well in controlling the outbreak of COVID-19, such as Singapore. Informational and unofficial contact tracing apps were excluded from this study. A total of 30,000 reviews corresponding to the 13 apps were scraped from app store webpages and analyzed.

**Results:**

This study identified seven distinct technologies used by COVID-19 tracing apps and 13 distinct apps. The United States was reported to have released the most contact tracing apps, followed by Italy. Bluetooth was the most frequently used underlying technology, employed by seven apps, whereas three apps used GPS. The Norwegian, Singaporean, Georgian, and New Zealand apps were among those that collected the most personal information from users, whereas some apps, such as the Swiss app and the Italian (Immuni) app, did not collect any user information. The observed minimum amount of time implemented for most of the apps with regard to data destruction was 14 days, while the Georgian app retained records for 3 years. No significant battery drainage issue was reported for most of the apps. Interestingly, only about 2% of the reviewers expressed concerns about their privacy across all apps. The number and frequency of technical issues reported on the Apple App Store were significantly more than those reported on Google Play; the highest was with the New Zealand app, with 27% of the reviewers reporting technical difficulties (ie, 10% out of 27% scraped reviews reported that the app did not work). The Norwegian, Swiss, and US (PathCheck) apps had the least reported technical issues, sitting at just below 10%. In terms of usability, many apps, such as those from Singapore, Australia, and Switzerland, did not provide the users with an option to sign out from their apps.

**Conclusions:**

This article highlighted the fact that COVID-19 contact tracing apps are still facing many obstacles toward their widespread and public acceptance. The main challenges are related to the technical, usability, and privacy issues or to the requirements reported by some users.

## Introduction

### Overview

The COVID-19 pandemic, the virus of which causes a highly contagious respiratory infection, has spread rapidly across the world and surpassed 20 million cases by early August 2020 [1]. The economic impact of the pandemic is felt globally with many countries slipping into recession. The COVID-19 pandemic is also turning into a job crisis, which is threatening to dismantle several industries, from aviation and manufacturing to services, tourism, and agriculture [2].

The global public health and government responses to the pandemic have been fragmented due to the urgency of actions required as a result of the stochastic spread of the virus. Some countries are implementing policies to eradicate the virus, such as Vietnam and New Zealand [3]; some countries are trying to suppress and contain the spread of the virus, such as Australia [4]; and some countries are relying on building herd immunity, such as Sweden [5]. Nonetheless, the virus continues to spread arbitrarily between regions and countries, and the epicenter of the pandemic has been moving between continents. It started with China and moved to Italy, Spain, the United States, and Brazil, with India as the next in line. Several other countries are now experiencing a second wave after initially suppressing it, with clusters of new cases popping up in many countries [6].

The speed of the authorities’ response has also proven to be a major key in containing the spread of the virus. For instance, many experts weighed in on the relatively slow response of Italy to contain the virus [7] and the fast response of South Korea in suppressing it [8]. Despite the variations in the worldwide governmental crisis responses to the pandemic and the lack of clear and uniform advice on matters as simple as the role of a mask in containing the spread of the virus [9], the measures and policies used worldwide to contain the virus remained mostly precautionary in the absence of a vaccine or a treatment. Consequently, the direct safety advice as a result of the COVID-19 pandemic continues to be about maintaining good hand hygiene, practicing social distancing between people, testing as soon as virus symptoms appear, quarantining, and, importantly, contact tracing.

Contact tracing is the process of identifying, assessing, and managing people who have been exposed to a disease to prevent onward transmission [10]. Until a COVID-19 vaccine is commercially available to the public, contact tracing tools are vital in breaking the chains of transmission of the virus. This means identifying infected people and their close contacts, testing them, and isolating them for 14 days from day zero of the exposure. For countries that managed to control the exponential growth of the virus, known as flattening the curve, extensive contact tracing was essential in minimizing large-scale community transmissions. With countries recently coming out of lockdown and opening their economies and borders again, such as France and the United States, contact tracing is the key to rapidly identifying new cases; hence, maintaining low levels of community transmissions to remain successful in containing the outbreak of the virus. Thus, in addition to comprehensive testing capacity, contact tracing is increasingly becoming important in managing this pandemic until a vaccine or a reliable viral treatment is successful and made publicly available.

For contact tracing to be beneficial in preventing onward transmission, and thereby reducing the impact of a second wave of a contagious disease such as COVID-19, it should be implemented systematically. This means having a system to securely collect, compile, and analyze data about individuals in real time, while not impinging on their privacy. As with the lack of a uniform and standardized global response to the pandemic, contact tracing technologies and approaches adopted by several countries were also diverse. For instance, on the same day in which Canada announced that they were working on a new contact tracing app [11], the United Kingdom was abandoning their contact tracing app, stating that the technology does not work [12].

### Background

Contact tracing using a mobile app relies on the concept of proximity tracking. The concept behind contact tracing is to identify and keep a record of people who may have been in close proximity (eg, typically less than 1.5 meters) to other people. Therefore, once an individual is identified to be infected with COVID-19, the app will be used to retrieve and trace the other close contacts. There have been various implementations for contact tracing apps, and a range of technologies, security, and privacy approaches have been adopted across the globe. Notably, the effectiveness of these contact tracing technologies remains to be seen. More evidence is required to demonstrate whether these tools were successful in contact tracing and to determine their usefulness.

Before the COVID-19 pandemic, a range of digital and mobile health tools had been utilized for the purposes of infectious disease control and public health interventions. Aba et al [13] illustrated the variety of functionalities provided by mobile apps to mitigate the spread of the Ebola virus in Africa, ranging from contract tracing to surveillance to case management. Mobile apps have also had a similar range of success in the use of public health interventions mitigating the spread of tuberculosis in Botswana [14].

The current contact tracing apps for COVID-19, which have been widely used by several countries, mostly use Bluetooth as the underlying technology for proximity sensing. In an effort to contribute toward having a unified solution for contact tracing and to counter the limitations of using Bluetooth on the iOS platform [15], Apple and Google have also recently released a new framework to support contact tracing [16]. However, apps that implement this framework have not matured enough yet. Nonetheless, surveying the current apps in use; analyzing their privacy features, penetration, and intake; and measuring their reception by the public, including the ensuing issues faced, have not been fully explored. This is demonstrated in a survey of the prior literature, which is presented in Table S1 from Multimedia Appendix 1. We briefly summarize a review of the main studies from the literature below.

The user acceptability of contact tracing apps in five countries hit by the pandemic using a survey were investigated in Altmann et al [17]; however, the study did not review specific contact tracing apps. Similarly, several studies [18-22] did not review current COVID-19 apps through direct access of app stores. For instance, the work reported in Anglemyer et al [18] did a meta-analysis on medical databases to review contact tracing apps. Others used various methodologies to conduct their reviews.

Other works, such as the one reported in Collado-Borrell at al [23], attempted to identify smartphone apps that aimed to address the COVID-19 pandemic and analyzed their characteristics. However, the study did not investigate any specific app. It only classified the apps under specific categories, such as health, fitness, or medicine. The main security and data protection aspects relating to digital contact tracing frameworks and apps were also investigated in Martin at al [24]; the paper analyzed some of the privacy aspects, such as personal information access, data retention, and location tracking. The paper also highlighted some of the app’s public penetration; however, the study was only limited to apps from Google Play.

An overview of mobile apps being currently used for COVID-19 and their assessment using the Mobile Application Rating Scale was reported in Davalbhakta et al [25]. This study was limited only to India, the United States, and the United Kingdom. Other works, such as the one reported in Vaudena [26], studied Bluetooth-based contact tracing solutions, including Decentralized Privacy-Preserving Proximity Tracing (DP-3T) and Temporary Contact Numbers (TCN) protocols, from a centralized versus decentralized point of view. As such, the vulnerabilities and the advantages of both solutions were systematically reviewed. The work focused more on the underlying architecture used and the level of privacy protection each one presented; however, it did not review specific implementations of COVID-19 apps. Only a few apps were used to represent the centralized and decentralized approaches.

The work in Magklaras and Bojorquez [22] surveyed the data regulations and technology protocols relating to COVID-19 contact tracing apps. It also provided mapping for the global deployment of the COVID-19 contact tracing apps. The paper also discussed the challenges, including some privacy aspects, relating to Bluetooth-based contact tracking technologies. The work reported in Li and Guo [27] provided an in-depth review of COVID-19 tracing app technologies and processes, including app installation and registrations, encounter data processing and communication, and notifications. The paper also analyzed the security aspects of contact tracing app architectures (ie, centralized, decentralized, and hybrid) by assessing their risk against common security attacks, such as denial of service and carryover attacks. This paper as well as an additional review paper [28] discussed some users’ common concerns, but it did not qualitatively analyze any users’ reviews.

### Study Aims

To this end, the work presented in this paper reviews and evaluates most categories of COVID-19 contact tracing mobile apps in use today. To our knowledge, this is the first research study that primarily investigated the public’s and users’ perceptions of COVID-19 contact tracing apps. This study also aimed at studying the privacy feature implementations and the level of penetration these apps achieved. In extension to the first aim, we aimed to determine the outreach of the collated apps in terms of number of downloads, as reported not only by the app stores but also by the authorities of each of the apps’ corresponding countries. This is in addition to providing a quantitative overview of the common complaints suggested by app users in connection to privacy, battery drainage, technical difficulties, bugs, crashes, and more. Additionally, in relation to the second aim, the underlying apps’ architecture and associated aspects, such as how the communication or handshake between two devices in proximity took place and then how close contacts were reported, were also analyzed. We also investigated the timeline of when these apps were introduced. Lastly, extending from the third aim, we attempted to understand the nature, type, and extent of data capture of the apps, such as granularity of data that was captured (ie, location, identification, and accomplices), duration of data retention, option to discard and delete records, and whether opt-out options were provided to the user without uninstalling the app.

## Methods

### Selection of Apps, User Intake, and Penetration

This study classified contact tracing apps based on the type of technology used for contact tracing of infected masses. This study identified six distinct technologies and an additional category commonly used or incorporated into COVID-19 tracing apps. These included Bluetooth, the DP-3T protocol, GPS, Pan-European Privacy-Preserving Proximity Tracing (PEPP-PT), the TCN protocol, Google and Apple, and other technologies, mainly the use of Quick Response (QR) codes paired with a digital diary. These technologies are outlined in Table S2 from Multimedia Appendix 1.

The classification criteria considered the underlying technology used by the apps rather than classifying the apps based on geographical or other architectural features. This is because most of the apps in use today use Bluetooth. Therefore, classifying the apps based on the underlying technology ensures that the research is capturing most contact tracing solutions in use. For instance, contact tracing solutions used by Singapore, Australia, and Malaysia use the same technology (ie, BlueTrace). As such, there is little benefit to the research from surveying all three of these apps.

**Figure 1:**
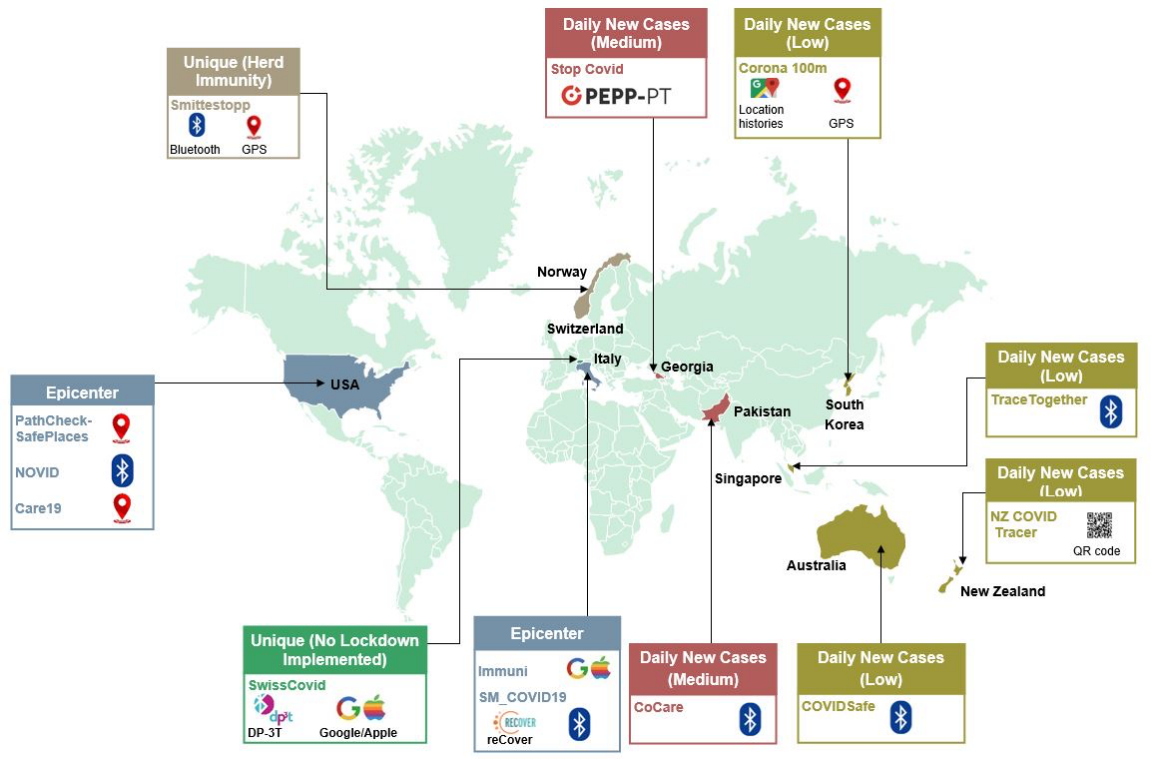
The 13 apps corresponding to 10 countries included in this study. DP-3T: Decentralized Privacy-Preserving Proximity Tracing; PEPP-PT: Pan-European Privacy-Preserving Proximity Tracing; QR: Quick Response.

Therefore, the research evaluated 13 apps corresponding to 10 countries and covered all the contact tracing technologies identified above. All apps were free to download. The inclusion criteria also ensured that most of the COVID-19-declared epicenters (ie, countries) were included in the sample, such as Italy and the United States. The evaluated apps also included countries that did relatively well in controlling the outbreak of COVID-19, such as Singapore; countries that had a low daily number of new infections (ie, Australia); and countries that had a medium-level daily number of new infections (ie, Pakistan). The Swiss app was included in this study, as Switzerland was among the few countries that did not implement a lockdown. Similarly, the Swedish app was also included, given Sweden’s unique approach to building herd immunity to combat COVID-19. Informational apps or unofficial contact tracing apps were excluded from this study, except for South Korea’s Corona 100m app, which uses GPS technology for contact tracing. This app was included because Corona 100m was among the first major contact tracing apps that launched across the globe and because South Korea is one of the few countries that managed to quickly suppress the transmission of the virus. Figure 1 shows the apps that were included in this study.

Table 1 details the architecture and approaches used by each of these technologies; these are as follows:

1. Country: For each type of technology used, a sample of countries that use this technology and their contact tracing apps are provided. Where there is more than one app used in a country, the name of the corresponding app is provided. It is worth noting that this is not a comprehensive list. The aim is to evaluate some of the countries for the purpose of adding context to the data presented in the table rather than creating an inventory of apps. The next section provides more details on the selection and inclusion criteria of the apps evaluated in this study.
2. Architecture: This criterion investigates whether or not the technology used by the contact tracing app incorporates the concept of uploading contact logs to a central reporting server. The criteria used are *centralized*, *semicentralized*, and *decentralized*. It has proven difficult to exclusively classify the architecture of each of these technologies, as implementations varied from one app to another. For instance, some apps uploaded contact logs to a central server, but the server did not have access to the uploaded contact logs, nor was it responsible for any further contact tracing processing, while others had access. As such, this criterion should be read in conjunction with the other criteria presented in Table 1, mainly the *encounter handshake* and *infection reporting* criteria.
3. Encounter handshake: This refers to how two devices coming into close contact perform a handshake (ie, exchange identification data). Most of the technologies surveyed exchanged some form of a temporary ID, while others exchanged some form of a unique identifier that was either encrypted or in plain text, which also depended on the specific implementation of each of the apps.
4. Infection reporting. This refers to how the contact log is reported to the central server and the role of this server in contact tracing. Most of the apps relied on the users to upload the contact logs. Implementations varied as well based on whether the health authorities had access to the contact logs or not.
5. Privacy by design. As the name suggests, this criterion explored whether the technology embedded any privacy considerations into its design specifications.

To analyze the users’ intake of each of the 13 apps under review and the penetration by these apps, this study extracted the following data for each of the apps: the name and country where the app was launched, the number of installs as per Google Play and as reported by the local news in the home country of the app, the penetration percentage as per Google Play installs and as reported by local news sources, and the launch date of each of the apps.

The number of installs were not only sourced from Google Play but also from local news outlets from the home country of each of the corresponding apps. The penetration percentages sourced from Google Play and the ones extracted from local news sources were calculated by dividing the total number of installs by the total population of the home country.

**Table 1:** The technologies of the contact tracing apps and their salient features.

App information	App technology						
	Bluetooth	DP-3T^a^	GPS	PEPP-PT^b^	TCN^c^	Google and Apple	Others
Example countries^d^	Australia; Singapore; Malaysia (MyTrace)	Austria; Finland; The Netherlands	Iceland (Ranking C-19); Italy (Diary); Jordan (Aman)	France; Georgia; Italy (Immuni)	Germany (ITO); Italy; The United States	Canada; Switzerland (SwissCovid); Germany (Corona-Warn-App)	New Zealand (digital diary); Australia, Canada, and New Zealand (GetHomeSafe); Malaysia (SELangkah)
Architecture	Centralized	Decentralized	Centralized	Centralized	Semicentralized	Decentralized	Centralized
Encounter handshake	Users exchange temporary IDs issued by the server	Unique 128-bit pseudorandom identifier by the server	Varies by implementation; some identify users by phone numbers	Users exchange temporary IDs issued by the server	TCN	Unique identifiers that are encrypted with a secret daily key held by the sending device	Real ID
Infection reporting	User-triggered upload	User-triggered upload, but the health authority never has access to contact log	User-triggered upload	User-triggered upload, but received massive privacy backlashes	The app notifies the user to potential infection	Not provisioned; delegated to app implementation	Varies by implementation; mostly user triggered
Privacy by design	No	Yes	No	No	Yes	Yes	No

^a^DP-3T: Decentralized Privacy-Preserving Proximity Tracing.

^b^PEPP-PT: Pan-European Privacy-Preserving Proximity Tracing.

^c^TCN: Temporary Contact Numbers.

^d^Where there was more than one app used in a country, the name of the corresponding app is provided within parentheses.

### Investigating the Privacy-by-Design Features and Privacy Implementations of COVID-19 Contact Tracing Apps

This study expands on previous work [29] that compared the privacy aspects of the COVIDSafe app (Australia, Bluetooth) and the COVID Tracer app (New Zealand, QR code). Each of the selected apps was downloaded and evaluated thoroughly. The study first identified the underlying technology used for contact tracing by an app and the amount of personal information each app collected (ie, personal information access). To do that, the following scale was used: if an app was only collecting the name, email, and phone number of the user, then the scale was designated as *low*; if, in addition to this personal information, the app collected the age of the user, then the scale was designated as *medium*; and if an app collected the name, email, phone number, age, and any additional information, such as the address, ethnicity, or location via GPS of the user, then this criterion was rated as *high*.

Additionally, the study analyzed the location features and tracking capabilities for each of the apps. It investigated whether an app was tracking the movement of individuals or not (ie, location tracking). It also investigated whether the app under review knew the identity of the people in close proximity to the user or just their locations or IDs (ie, true identity vs temporary ID, such as with the TCN protocol). The criterion *tracking and identifying proxies* combined the *encounter handshake* and *infection reporting* features.

Furthermore, this study investigated the record-keeping time frame of each app. This was achieved by researching the duration that the contact logs were kept on the device or the authority’s remote servers for each of the apps under review.

In terms of user control, this study examined two criteria: the user’s right to forget and the geo-restrictions imposed on an app. The first criterion considered whether or not users were informed about the procedures to delete the records collected by an app. The opting-out criterion explored whether the users were able to sing in and out of the app under review. Lastly, for each app under review, the study investigated whether an app could be downloaded from anywhere or whether it was a home or region geo-restricted app (ie, geo-restriction). We referred to governments’ media releases, white papers, and developers’ announcements for the apps that were in testing phases or were not available on the Apple App Store or Google Play.

### Analyzing the Public Reception of COVID-19 Contact Tracing Apps

We aimed to identify the audience uptake and users’ feedback of the COVID-19 contact tracing apps under review. Data were sourced by scraping the publicly available user reviews from the Apple App Store and Google Play webpages for each of the apps. Scraping is a process or tool used to extract data from a website; in this case, reviews from Google and Apple stores. Almost 30,000 reviews were scraped and analyzed in this study. The user reviews of each of the corresponding apps were then filtered and analyzed using a brute-force keyword search methodology; this means extracting the user reviews that contained a specific keyword used in the search. Table 2 lists the keywords used in scraping the reviews. The methodology used for analyzing these reviews also accounted for the variations of each of the keywords, referred to as subkeywords. For instance, the results of scraping and analyzing certain subkeywords—*doesn’t work*, *didn’t work*, *not working*, *Doesn’t work*, *Didn’t work*, and *Not working*—were all counted toward the results of the main keyword *Malfunctioning*. In other words, the results reported under the keyword *Malfunctioning* are a concatenation of each of the individual results returned by its list of subkeywords.

**Table 2:** The keywords used in this study.

Keywords	Subkeywords
Drainage	drain; battery; Drain; Battery
Spyware	spy; spied; spyware; Spy; Spied; Spyware
Malfunctioning	doesn’t work; didn’t work; not working; Doesn’t work; Didn’t work; Not working
Crashes	crash; freeze; Crash; Freeze
Privacy concerns	privacy issue; privacy concern; location concern; tracking me; track me; tracking us; Privacy issue; Privacy concern; Location concern; Tracking me; Track me; Tracking us
Ineffective	useless; rubbish; garbage; Useless; Rubbish; Garbage
Bugs	bug; buggy; Bug; Buggy
Installation issues	can’t install; doesn’t install; couldn’t install; Can’t install; Doesn’t install; Couldn’t install
Incompatible	can’t download; couldn’t download; incompatible; Can’t download; Couldn’t download; Incompatible

## Results

### Selection of Apps, User Intake, and Penetration

In this section, we initially describe results on app penetration. A challenging aspect of sourcing the data reported in Table 3 [30-40] was encountered when calculating the intake of the apps under study. For instance, the number of downloads for an app does not represent the true value of the actual intake. Downloading an app does not necessarily mean the app is being used. Users may simply download the app and never use it or uninstall it. In addition, there were little data available on the number of uninstalls for each of the surveyed apps. Regardless of this limitation, the number of installations for an app was not available on the Apple App Store. This has made the task of calculating the uptake of an app even more complex.

Consequently, the research required access to a more precise estimate of the installation values as compared to what Google Play was showing. Therefore, apart from consulting Google Play’s number of installs, the study referred to reliable news sources to obtain the total number of registrations or downloads for each of the apps under review. The news sources were mainly from government or developer announcements, verifiable local news sources, and published research (ie, white papers). Some of the statistical information, such as the download intakes and any data sourced from local news, was available as of early July 2020. As such, there might be a slight variation in the values presented in Table 3 as compared those at the time of the archiving of this paper. Some apps were new, so this local value was not readily available for those either. Another challenge this research study ran into was the unavailability of some of the apps on the Google Play Store. This is because they were discontinued or because they were still in demo or beta stages.

**Table 3:** Penetration and intake of the 13 selected contact tracing apps.

Country	App	No. of installs, n		Penetration, %		No. of days of the app's launch since patient zero^a^, n
		Local news	Google Play Store^b^	Local news	Google Play Store^b^	
United States	PathCheck SafePlaces	N/A^c^	10,000	N/A	0.001	93
United States	NOVID	N/A	10,000	N/A	0.001	110
United States	Care19	33,000 [31]	10,000	0.01	0.001	76
Italy	Immuni	2,700,000 [32]	1,000,000	4.47	1.65	122
Italy	SM-COVID-19	52,000 [33]	50,000	0.09	0.08	73
Norway	Smittestopp	1,427,000 [34]	100,000	26.32	1.84	50
Singapore	TraceTogether	2,100,000 [35]	1,000,000	35.89	17.09	57
South Korea	Corona 100m	1,000,000 [36]	N/A	1.95	N/A	20
Pakistan	CoCare	N/A	500	N/A	0.001	108
Australia	COVIDSafe	6,130,000 [37]	1,000,000	24.03	3.92	91
New Zealand	NZ COVID Tracer	573,000 [38]	100,000	11.88	2.07	82
Switzerland	SwissCovid	1,600,000 [39]	500,000	18.48	5.78	90
Georgia	Stop Covid	100,000 [40]	100,000	2.51	N/A	50

^a^The first case in the country was reported on the Johns Hopkins Coronavirus Resource Center portal [30].

^b^All download values have been extracted from the app’s webpage on Google Play.

^c^N/A: not applicable; data for apps were unavailable because the app was new, in the case of local news, or because it was discontinued or was still in demo or beta stages, in the case of the Google Play Store.

The results of this study show that South Korea was the first country to use a mobile app for contact tracing during the COVID-19 pandemic. The South Korean app, Corona 100m, was only introduced after 20 days from the first detected case of COVID-19 in South Korea. This was followed by Singapore, Norway, and Georgia, which introduced their apps around 50 days since patient zero. The United States, Italy, and Pakistan were slower, as they introduced their contact tracing app around the 100-day mark. As reported by local news, the Singaporean (36%) and Norwegian (26%) apps had the highest penetration intake, followed by the Australian and Swiss apps, which had around 20% penetration, and the New Zealand app, which achieved around 11% penetration. Interestingly, the Italian and US apps had the lowest penetration values. The penetration intake of the apps on the Android platform, which was calculated based on the Google Play–reported number of installs for each of the apps, showed that all apps under review, except for the Singaporean app, had very poor intake (<5%).

Furthermore, this research initially intended to calculate the success rate of each of the apps in contact tracing reporting. It also aimed to survey and compare the efficacy of the apps under review. However, this was challenged by the lack of any reliable relevant data available in relation to those aspects; thus, this part of the review had to be excluded. Therefore, it is unclear whether the early introduction of contact tracing apps has contributed toward their rapid public adoption or whether these apps have played a major role in the contact tracing efforts of COVID-19. Perhaps these apps have played some part in raising awareness among the public, as in the case of the Singaporean and Australian apps, which had higher penetration intake values and lower infection numbers comparatively. However, due to insufficient data, no conclusive results can be made on the correlation between the early introduction of a contact tracing app, its higher penetration intake, and the case where low number of COVID-19 transmissions were reported.

### Investigating the Privacy-by-Design Features and Privacy Implementations of COVID-19 Contact Tracing Apps

In the subsequent sections in this paper, when referring to an app, the following notation shall be used: app name (country of origin, technology used for contact tracing).

Table S3 from Multimedia Appendix 1 reviews the privacy features of the 13 apps evaluated in this study. Each of these apps was downloaded and evaluated thoroughly as per the criteria shown in Table S3 from Multimedia Appendix 1. The research also referred to white papers and developers’ announcements for the apps that were in their testing phases or were not available or accessible on the Apple App Store and/or Google Play. The same methodology was followed for the apps that were not available in English, such as Immuni (Italy, Google and Apple application programming interface [API]) [41], SM-COVID-19 (Italy, ReCoVer) [42], and Smittestopp (Norway, Bluetooth and GPS) [43].

Nine of the apps were available for free on both the Apple App Store and Google Play. Two apps—SM-COVID-19 (Italy, Google and Apple) and CoCare (Pakistan, Bluetooth) [44]—were only available on Google Play, while Stop Covid (Georgia, PEPP-PT) [45] was only available on the Apple App Store. The Corona 100m app (South Korea, location) [46] was not available on both stores. Smittestopp (Norway, Bluetooth and GPS) was not available to download due to geo-restrictions. The Australian COVIDSafe app required an Australian phone number and a postcode to run.

Bluetooth was the most frequently used underlying technology, employed by seven apps for digital contact tracing, whereas three apps performed contact tracing through location (eg, GPS). The apps using location as the underlying technology, namely Corona 100m (South Korea, location) and PathCheck SafePlaces (United States, location), tracked and recorded the locations visited by the users. Although Corona 100m (South Korea, location) was removed from Google Play, the app integrated GPS history, data from nationwide surveillance cameras, and credit card transactions. This has sparked privacy concerns, as users of the Corona 100m app could see the date when a COVID-19 patient was infected, along with his or her nationality, gender, age, and the locations they visited.

The Norwegian, Singaporean, Georgian [45], and New Zealand [47] apps were among the apps that collected the most personal information from the users, while some other apps, such as the Swiss app [48] and the Italian Immuni app, did not collect any user information. Other apps ranged from simply collecting users’ phone numbers to additionally collecting their names or email addresses.

Data destruction was incorporated into most of the apps, which automatically deleted the users’ records after 14 days, the observed minimum amount of time implemented in most of the apps. Some kept these records for 21 days (ie, Australia) and others for 30 days (ie, Switzerland and India); the New Zealand app kept them for 31 days, while the Georgian apps kept them for 3 years, the longest of any app.

Three of the US apps—PathCheck (United States, location) [49], NOVID (United States, Bluetooth radio waves and ultrasound) [50], and Care19 (United States, GPS) [51]—did not require users to sign up before using their app. On the other hand, many apps, such as the Singaporean TraceTogether app [52], the Australian COVIDSafe app [53], and the Swiss and Indian apps, did not provide the users with an option to sign out from their app. It is noteworthy to mention that the data presented in Table S3 of Multimedia Appendix 1 are accurate as of June 30, 2020.

### Analyzing the Public Reception of COVID-19 Contact Tracing Apps

**Figure 2:**
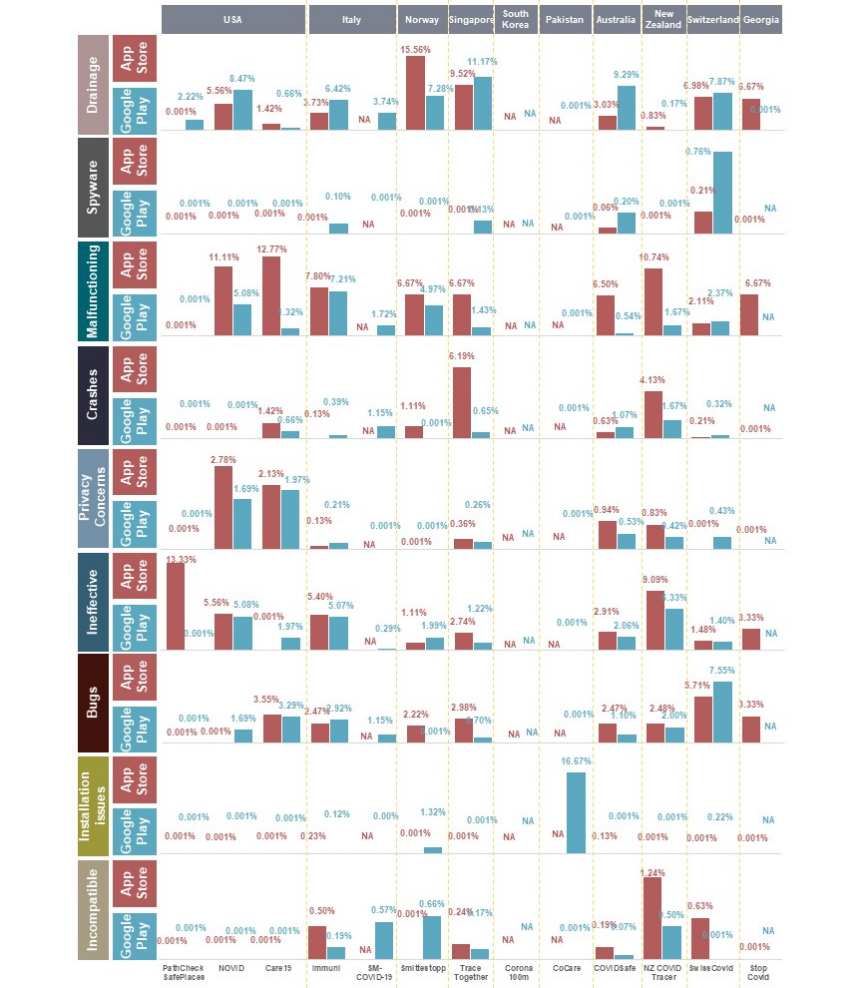
Percent occurrence of each keyword for each app. NA: not applicable; user reviews were unavailable, as the corresponding apps were not available on the corresponding platforms.

**Figure 3:**
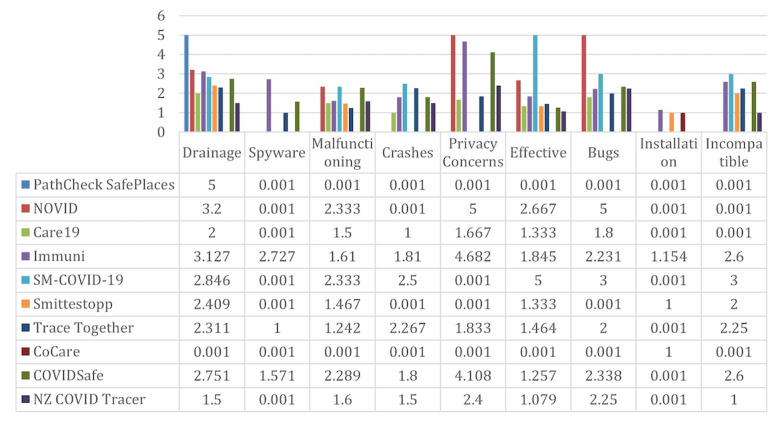
Average ratings out of 5 stars from user reviews in each category of each app on Google Play.

Figure 2 shows the percent occurrence for each of the keywords for each app. Figure 3 shows the average ratings of the reviews for each keyword. For example, consider if a user left a review for one of the apps saying, “the app keeps on crashing,” and then gave it a rating of 2 stars. This review will then be counted toward the average mentions of the keyword *crashes* shown in Figure 2. The 2-star rating will also be counted toward the corresponding keyword average rating shown in Figure 3. All small values were rounded up to 0.001.

Three of the applications—CoCare (Pakistan, Bluetooth), SM-COVID-19 (Italy, ReCoVer), and Corona 100m (South Korea, location)—were not available on the Apple App Store, whereas two apps—the Corona 100m (South Korea, location) and Stop Covid (Georgia, PEPP-PT)—were not available on Google Play. Based on the frequency of keyword occurrences, *Drain*, *Malfunctioning*, and *Ineffective* were the most frequent issues reported by the users in their reviews.

On the Apple App Store, the keyword *rubbish* had a 13.33% occurrence for PathCheck SafePlaces (United States, location), 5.56% for NOVID (United States, Bluetooth), 5.40% for Immuni (Italy, Google and Apple API), and 9.09% for NZ COVID Tracer (New Zealand, digital diary). Similarly, many users did not find contact tracing apps functional. On the Apple App Store, many app users complained that their app did not work. This was represented by the keyword *Malfunctioning,* which had a 10.74% occurrence for NZ COVID Tracer (New Zealand, digital diary), 6.50% for COVIDSafe (Australia, Bluetooth), 6.67% for TraceTogether (Singapore, Bluetooth), 7.80% for Immuni (Italy, Google and Apple API), 11.11% for NOVID (United States, Bluetooth), and a sharp 12.77% occurrence for Care19 (United States, Apple and Google). Many users also had problems with the apps’ compatibility with their operating system and frequent crashes. For instance, CoCare (Pakistan, Bluetooth) had a 16.67% occurrence for the incompatibility issue.

**Figure 4:**
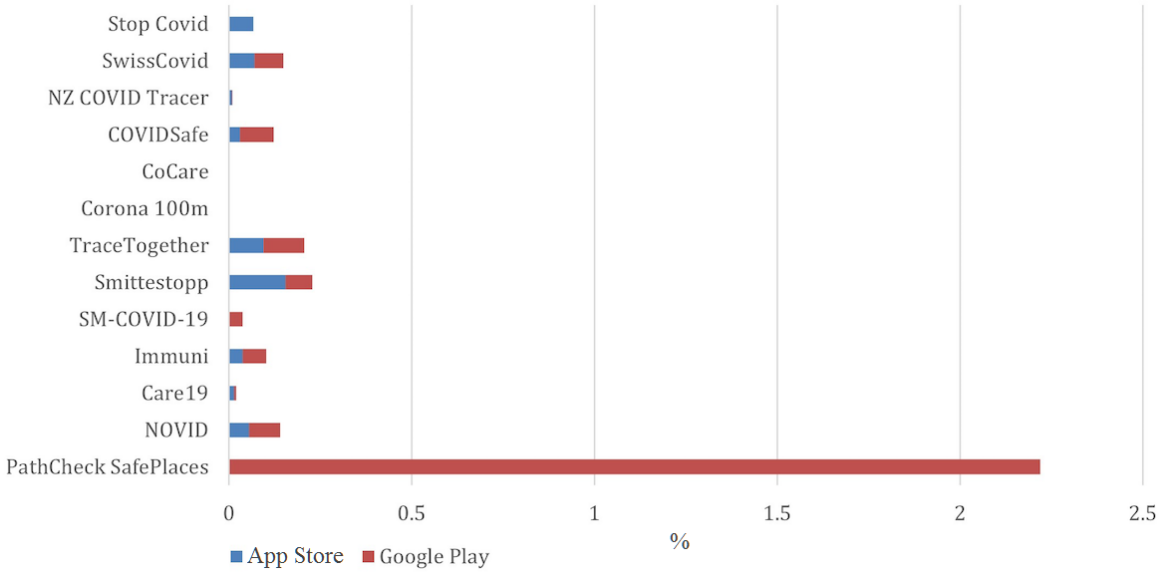
Percent occurrence of the keyword "drainage," pertaining to battery drainage.

**Figure 5:**
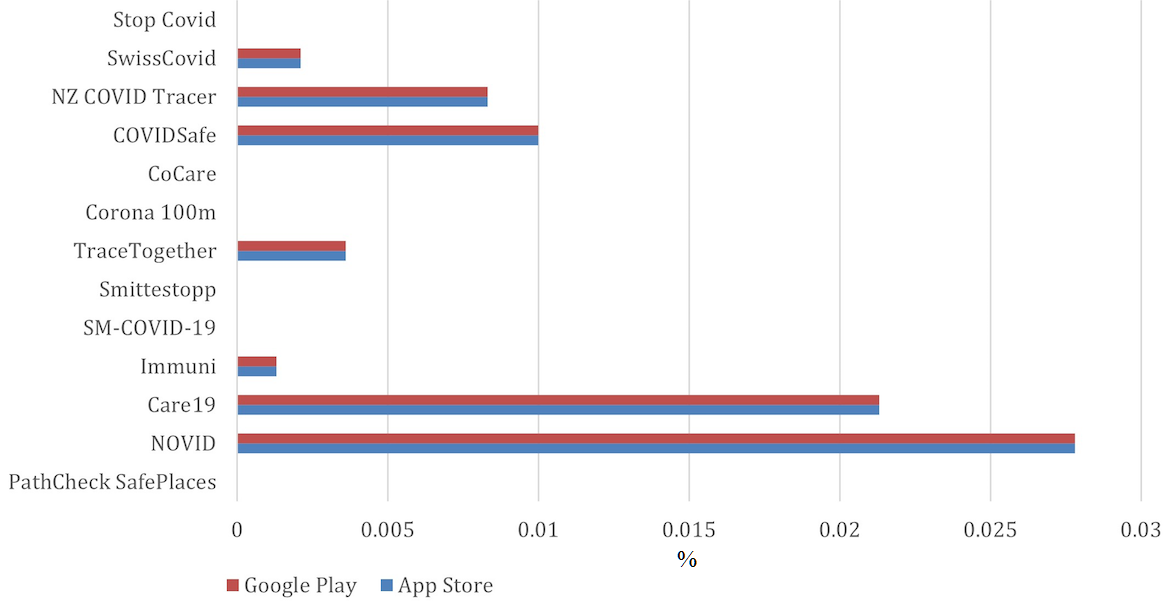
Percent occurrence of the keywords "privacy concerns" and "spyware".

Interestingly, and as shown in Figure 4, no significant battery drainage issue had been reported for most of the reviewed apps. The privacy concerns reported by the users were also very minimal across all apps, as shown in Figure 5.

**Figure 6:**
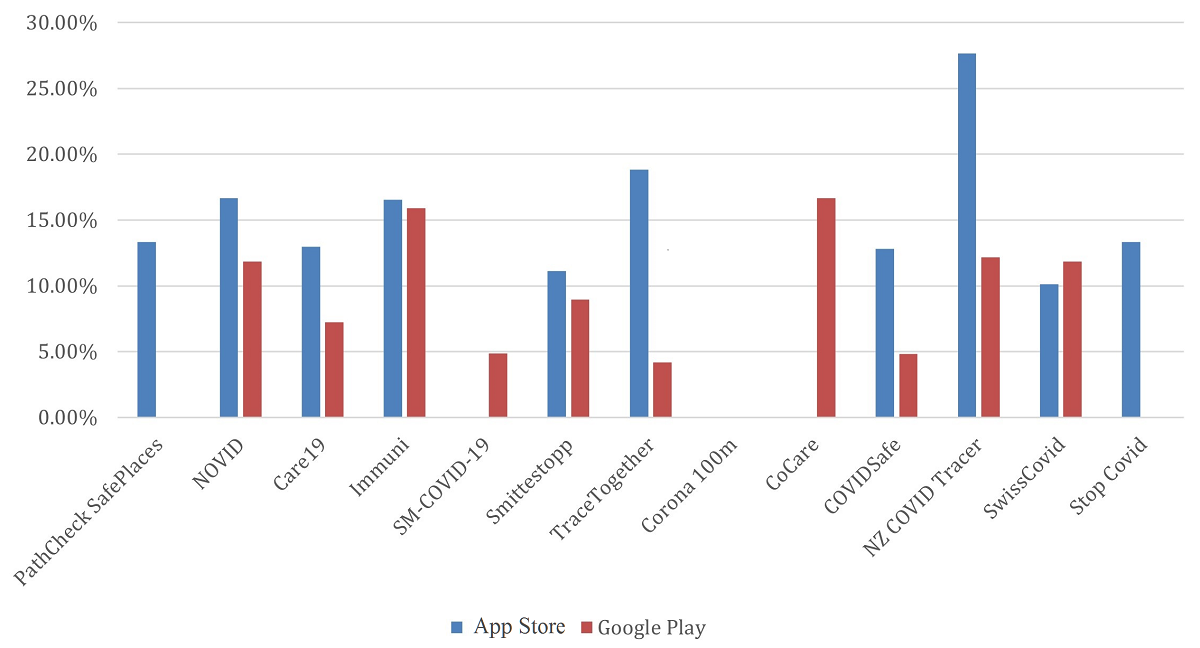
Summary of technical issues reported for each of the apps. The plot shows results from the combination of the following keywords, along with their respective subkeywords: "Malfunctioning," "Crashes," "Ineffective," "Bugs," "Installation issues," and "Incompatible".

**Figure 7:**
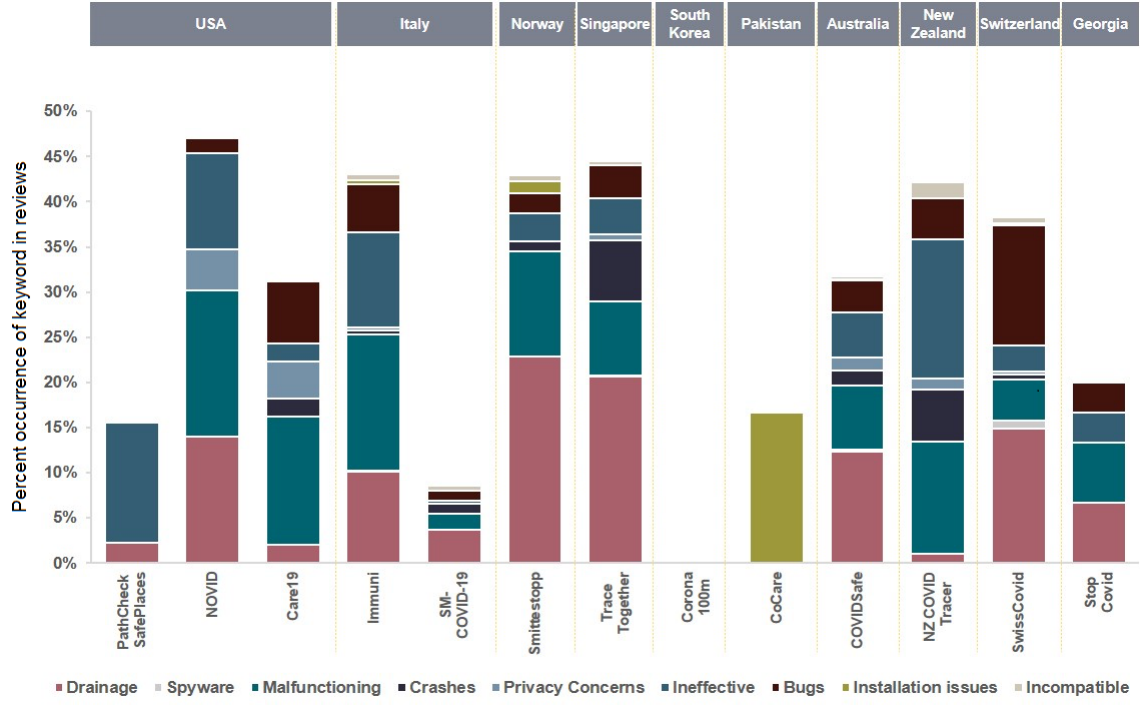
Comparison of user app reviews and their inclusion of various keywords.

Figures 6 and 7 provide overall insights into the technical issues reported by the users for each of the apps. These figures combine the results of the following keywords, along with their respective subkeywords: *Malfunctioning, Crashes, Ineffective, Bugs, Installation issues, and Incompatible*. It is obvious that most apps on the Apple App Store had the most reported technical issues when compared to their Google Play counterparts, except for the Swiss contact tracing app. The US PathCheck app had the least reported technical issues on Google Play, while the New Zealand app version on the Apple App Store had the most technical issues that were complained about across all apps and platforms.

## Discussion

### Principal Findings

Our research study has highlighted the hindrances in the successful deployment of COVID-19 contact tracing apps. The use of mobile technologies for contact tracing has been met with a number of challenges [54], many of which also emerged in the contextual evaluation of user reviews on COVID-19 contact tracing apps as described in our study. Among these, the most popular were technical malfunctions and drainage of battery.

Other challenges included privacy. Of course, this is anticipated, as you cannot expect to trace and track peoples’ movements by a government authority without addressing privacy issues [55]. Nonetheless, in addition to privacy, there were many other challenges and limitations hindering the anticipated efficacy from contact tracing apps.

For instance, a mobile contact tracing app needs to be widely adopted by a population for it to be of benefit; this is challenging to achieve. Penetration of COVID-19 contact tracing apps remains low despite governments pushing for mass use [56]. The widespread adoption of contact tracing apps requires that people would have access to a smartphone and, in most cases, access to a reliable internet connection. Hence, in countries with large populations, such as Pakistan [57], the smartphone penetration percentage sits at only 15%, and in Indonesia this value sits at only 31%. Users may also feel uncomfortable if there is no clear opt-out strategy [58]. Nearly half of the apps reviewed in our study did not provide a transparent withdrawal avenue.

Furthermore, the approaches used by contact tracing apps rely mostly on one single parameter (ie, proximity, such as via Bluetooth) [59]. However, proximity by itself is not enough to determine the risk of someone being exposed to the virus. There are a number of other parameters involved, such as being indoors or outdoors, being in a room with good air circulation or not, and the issue of surface infection exposure, irrespective of the proximity of an individual to an infected person. Furthermore, although as shown in our review that Bluetooth was one of the more popular technologies to implement contact tracing apps, every country’s regulations may differ [60]; hence, a one-size-fits-all approach may be problematic.

Other challenges pertain to the limitations associated with the technology used for contact tracing. For instance, the use of GPS as a proximity technology is not reliable in indoor environments [61]. Determining the distance between two persons using Bluetooth technology also has its own set of challenges, such as signal strength attenuation caused by some environmental factors (eg, if the phone is placed inside a thick pocket or if the phone is at an angle facing a wall).

Nevertheless, contact tracing technologies surveyed in this work have been found to use a locationless tracking approach; that is, the app does not trace or record people’s movements, obviously for privacy purposes. Therefore, most of these apps can only determine if two people were in proximity at a given time, but they do not keep a log of the users’ movements. Consider, for example, if an infected person, labelled as Pi, is in a supermarket and Pi touches an item at time t–1 at a location designated as Li. Another person who is not infected, designated as Pn, is at a location designated as Ln. There is no proximity between Pi and Pn. Now assume Pi leaves the store at time t, when at the same time (ie, at t) person Pn moves from Ln to Li. There is a high chance that Pn is going to be infected if they touch the same item Pi touched at t–1 (ie, surface infection exposure). To be able to capture this exposure, contact tracing apps require the use of a location-oriented tracking approach in which the locations and movements of people are compared against each other to determine the overlapped and colluded locations. Future work will explore the use of our already well-established location obfuscation technique [62] in a contact tracing solution. The work will aim at providing a location-oriented contact tracing app without impinging on users’ privacy.

### Limitations

One of the challenges encountered in scraping the reviews was analyzing the apps that were not available in English. For example, most of the reviews for the Immuni app (Italy, Google and Apple), SM-COVID-19 (Italy, ReCoVer), and Smittestopp (Norway, Bluetooth and GPS) were available in the Italian and Norwegian languages, respectively. For these reviews, along with the rest of the app reviews that were in different languages, the keywords along with their subkeywords were translated into the language of their home app country. The results were incorporated when calculating the overall average values for all the apps. The translated keywords along with the subkeywords used can be found in Table S4 from Multimedia Appendix 1. Another limitation in our methodology for review scraping lies in the presence of false negatives in some of the reviews. This is one of the limitations of brute-force keyword search methodology. Take, for instance, one of the reviews for COVIDSafe (Australia, Bluetooth) on Google Play:

Installed from its release. Worked. No problems at all. It doesn't drain the battery. It doesn't crash. It's totally fine. I haven't been dragged into the back of a van, taken to an underground bunker and questioned by spies.

The review is classified as a false negative for the words *drain* and *crash*. It can be debated that the number of false negatives could have been reduced by simply taking the *battery* subkeyword out from the keyword search (ie, battery; drain). However, in doing so, the number of 1-star reviews were significantly reduced by more than 50%. For instance, with NZ COVID Tracer (New Zealand, digital diary), the 1-star reviews dropped from 23 to 10 after taking the word *battery* out of the search filter. The reason behind this is that the users’ reviews were not systematic. Most users represented their opinions in natural language. Some samples of 1-star reviews for COVIDSafe (Australia, Bluetooth) commenting on the app’s drainage issue are as follows:

It is of no use whatsoever. A waste of money & a waste of my battery life.

Battery went from 100% to zero in 5 hours with not much use. I usually get a full day out of it.

Hard on the battery.

Therefore, for the sake of including these comments, the subkeyword *battery* was not removed from the keyword search results.

This study has a number of additional limitations. This paper is based solely on 13 evaluated apps. While the selection criteria ensured that apps were selected to represent each of the categories of technology used for contact tracing, it did not review all COVID-19 contact tracing apps. Also, the study relied on data that were extracted and accurate as of July 2020. Another major limitation of this work relates to the penetration intake calculations done for each of the apps. The study derived the percent penetration for each app by dividing the number of installs of an app by the population of the home country. This method suffers from several shortcomings. The number of downloads or installs cannot be precise; it ignores the fact that some users may install, uninstall, and reinstall the app several times or on more than one device owned by the user. A user may also download the app and never use it. Many users may not download an app yet may still post a review about it. For these reasons, the study attempted to gauge the penetration intake for each of the apps by analyzing the local government announcements and reports published by local news agencies. However, since the reported download values cannot be verified nor the methods used to derive them, the trustworthiness of these values also remains invalidated. Lastly, although the study evaluated about 30,000 user reviews, the reviews cannot not be verified.

### Conclusions

While public health agencies attempt to understand the efficacy of nonpharmaceutical interventions [63], contact tracing has been a key part of the worldwide measure in response to the COVID-19 pandemic. For contact tracing to work effectively, solutions such as tracing apps should be implemented systematically. This requires the secure collection, processing, storage, and discarding of contact tracing information of people in real time, without impinging on their privacy and rights. The success of contact tracing apps greatly depends on their large uptake within a population, in addition to strong public health enforcement. This article highlighted the fact that COVID-19 contact tracing apps are still facing many obstacles toward their widespread and public acceptance.

The main challenges are related to the technical, usability, and privacy issues or requirements reported by some users. This meant that most tracing apps were not publicly well-received and had low penetration levels, which hinders their effectiveness. For instance, only the Singaporean app had a penetration of slightly over 30%, the Australian and Swiss apps achieved penetration just below 20%, and the penetration values reported for most of the other apps were very poor, sitting at below 5%. The amount of personal data collected by the apps varied widely, with some apps not collecting data at all and others collecting a significant amount of sensitive data about the user, such as their ethnicity. The majority of the surveyed apps did not provide the user with options to opt out from the apps, such as logging out, without uninstalling them.

The lack of a standardized contact tracing approach also meant that contact tracing apps used across the globe were fragmented and noninteroperable. As most countries are now coming out of lockdown and reopening their borders, there is an increased need for a cohesive, cross-border, and interoperable contact tracing app that can be used universally without impinging on users’ privacy. Additionally, there is a lack of available data on the effectiveness of COVID-19 contact tracing apps. As we progressively recuperate from this pandemic, there is a need to re-evaluate and re-examine the values and roles of contact tracing apps in controlling infectious diseases such as COVID-19.

## Appendix

Multimedia Appendix 1 Summary of papers related to contact tracing apps, the main technologies used, the privacy features, and translated keywords.

## Abbreviations

API: application programming interface
DP-3T: Decentralized Privacy-Preserving Proximity Tracing
PEPP-PT: Pan-European Privacy-Preserving Proximity Tracing
QR: Quick Response
TCN: Temporary Contact Numbers
