# Supplementary material for: Technology, Privacy, and User Opinions of COVID-19 Mobile Apps for Contact Tracing: Systematic Search and Content Analysis
Source: J Med Internet Res. 2021 Feb 9;23(2):e23467. doi: 10.2196/23467 (PMC7879719; doi:10.2196/23467)
Supplement: Multimedia Appendix 1 [file jmir_v23i2e23467_app1.docx]

Table 1- Summary of related works

| **Paper** | **Privacy** | **Penetration** | **User Review** |
| --- | --- | --- | --- |
| Acceptability of App-Based Contact Tracing for COVID-19: Cross-Country Survey Study [17] | no apps are reviewed | no apps are reviewed | no apps are reviewed |
| Digital contact tracing technologies in epidemics: a rapid review [18] | no apps are reviewed | no apps are reviewed | no apps are reviewed |
| Features and Functionalities of Smartphone Apps Related to COVID-19: Systematic Search in App Stores and Content Analysis [23] | No | Only as per Google Play. | No |
| Digital technologies in the public-health response to COVID-19 [19] | no apps are reviewed | no apps are reviewed | no apps are reviewed |
| Demystifying Covid-19 Digital Contact Tracing: A Survey On Frameworks And Mobile Apps [24] | Partially | Only as per Google Play. | No |
| A Systematic Review of Smartphone Applications Available for Corona Virus Disease 2019 (COVID19) and the Assessment of their Quality Using the Mobile Application Rating Scale (MARS) [25] | No | No | No |
| Centralized or Decentralized? The Contact Tracing Dilemma [26] | Partially | No | No |
| Covid-19 And Contact Tracing Apps: A Review Under The European Legal Framework [20] | no apps are reviewed | no apps are reviewed | no apps are reviewed |
| Covid-19 contact tracing: a briefing [21] | no apps are reviewed | no apps are reviewed | no apps are reviewed |
| A review of information security aspects of the emerging COVID-19 contact tracing mobile phone applications [22] | no apps are reviewed | no apps are reviewed | no apps are reviewed |
| Global Deployment Mappings and Challenges of Contact-tracing Apps for COVID-19 [27] | Partially | Yes | No |
| A Survey of COVID-19 Contact Tracing Apps [28] | Partially | Partially | Partially |

Table 2- The main technologies used in contact tracing mobile apps.

| **Technology** | **Description** |
| --- | --- |
| Bluetooth | The subject’s phone uses proximity tracking in which encrypted tokens are exchanged with nearby phones via Bluetooth signals. The approach is easier to anonymize but comes with the challenge of signals’ attenuation. |
| DP-3T protocol | Decentralized privacy-preserving proximity tracking is an open protocol for contact tracing enabling full anonymity. It uses Bluetooth Low Energy for measuring a subject’s proximity. The subject’s phone’s contact logs and computation stay entirely on their device. The central reporting server nor has access to data, neither is responsible for processing information. This approach has major privacy benefits. |
| Location | The subject’s phone tracks their movements and looks for nearby phones in the same vicinity either by using GPS or triangulation from cell towers. This approach raises concerns for privacy-concerned users. |
| PEPP-PT/PEPP | Pan-European Privacy-Preserving Proximity Tracing, like DP-3T, relies on Bluetooth to discover and locally logs clients near a user. However, unlike DP-3T, this approach uses a central repository system to process contact logs. |
| TCN | Temporary Contact Numbers protocol is a decentralized and anonymous contact tracing protocol. It uses Bluetooth Low Energy to track and log encounters. As no central repository is involved in data collection and privacy, the protocol has huge privacy benefits. |
| Google/Apple | This is an API developed jointly by Google and Apple. Using the API, iOS and Android smartphone users communicate via Bluetooth. The protocol is highly influenced by DP-3T and TCN, but it is implemented at the operating system level. |
| Other | QR codes and digital diaries are used to log the locations visited by the users. |

Table 3- Privacy Features of the Reviewed Applications^[[1]](#footnote-1)^

| Country | App | Tech | Arch. | Loc. Track. | Loc Trac. of  proxies | Personal Information Access | Data Retention | Right to Forget | Optout | Geo Location Restricted |
| --- | --- | --- | --- | --- | --- | --- | --- | --- | --- | --- |
| USA | **PathCheck SafePlaces**  [49] | Location | D-Cen | Yes | No | Low  Encrypted location history is saved on phone | 14 days | No | No sign out feature | No |
|  | **NOVID** [50] | BT & ultrasound | Cen | No | No | Low  Require microphone permissions to receive ultrasound | 14 days | Yes  By uninstalling the app | No  However, user can disable the app | No |
|  | **Care19** [51] | GPS | Cen | Yes | Yes  Location associated with encrypted IDs | Low  Location data will only be shared if consented. | 14 days | Yes  Users can delete all data | Yes  Users can opt out anytime, | No |
| Italy | **Immuni** [41] | BT then moved to Google/Apple | D-Cen | No | No | Low  if consented | Until 31 Dec, 2021 | No | No sign out feature  User can disable the app. | No |
|  | **SM_COVID19**  [42] | ReCoVer, BT | Cen | Optional | No  Acquires ID of nearby devices | No | 21 days | If the APP remains installed | Yes  You can uninstall. | No |
| Norway | **Smittestopp** [43] | BT & GPS | Cen | Yes | No | Medium  Mobile phone number, age, GPS location, generated UUID Bluetooth data | 30 days | Yes | Yes | Yes |
| Singapore | **Trace Together**  [52] | BT | Cen | No | No | Medium  (Name, age, nationality, passport number) | 25 days | Yes  by contacting support | No | No |
| South Korea | **Corona 100m** [46] | Location histories | Cen | Yes | Yes | High  (age, sex, location. GPS history, data from nationwide surveillance cameras, and credit card transactions.) | N/A | N/A | N/A | App is no longer available.  info collected from white papers and articles [49] |
| Pakistan | **CoCare** [44] | BT | Cen | No | No  Only keys not locations | Low  (Mobile number) | 30 days | Yes  (By deleting the app) | Yes  you can logout of the app | N/A |
| Australia | **COVIDSafe** [53] | BT | Cen | No | Yes | Medium  (Name, phone, age, postcode) | 21 days | Yes | N/A | Yes  (postcode and number) |
| New Zealand | **NZ COVID  Tracer** [47] | BT, QR codes | Cen | Yes | No | High  (Name, email, address, phone, age, ethnicity, location) | 31 days | Yes  by deleting the app. | Yes | No |
| Switzerland | **SwissCovid** [48] | BT & DP-3T  Moved to Google/Apple | D-Cen | No | Yes  (the app does not record locations) | No | 14 days | Yes  by deleting the app | Yes  you can disable tracing or turn off Bluetooth | No |
| Georgia | **Stop Covid** [45] | PEPP-PT | Cen | Yes | No | Medium  User ID, Location data, Time, duration and location of the contacts, phone number | 3 years | No  historical information kept for maximum of 3 years. | N/A | No |

Table 4- Translated keywords

| italian | German | French |
| --- | --- | --- |
| drain \|\| batteria \|\| Drain \|\| Batteria | ablassen \|\| batterie \|\| Ablassen \|\| Batterie | batterie \|\| drainer \|\| Batterie \|\| Drainer |
| spiare \|\| spiata \|\| spiato \|\| spyware \|\| Spiare \|\| Spiata \|\| Spiato \|\| Spyware | spionin \|\| spion \|\| ausspioniert \|\| spyware \|\| spionin \|\| spion \|\| ausspioniert \|\| spyware \|\| Spionin \|\| Spion \|\| Ausspioniert \|\| Spyware \|\| Spionin \|\| Spion \|\| Ausspioniert \|\| Spyware | espionne \|\| espion \|\| espionné \|\| spyware \|\| Espionne \|\| Espion \|\| Espionné \|\| Spyware |
| non funziona \|\| non ha funzionato \|\| non funziona \|\| Non funziona \|\| Non ha funzionato \|\| Non funziona | funktioniert nicht \|\| hat nicht funktioniert \|\| funktioniert nicht \|\| Funktioniert Nicht \|\| Hat Nicht Funktioniert \|\| Funktioniert Nicht | ne fonctionne pas \|\| n'a pas fonctionné \|\| ca ne fonctionne pas \|\| Ne Fonctionne Pas \|\| N'a Pas Fonctionné \|\| Ca Ne Fonctionne Pas |
| schianto \|\| congelare \|\| Schianto \|\| Congelare | absturz \|\| einfrieren \|\| Absturz \|\| Einfrieren | crash \|\| geler \|\| Crash \|\| Geler |
| problema di privacy \|\| preoccupazione sulla privacy \|\| preoccupazione per la posizione \|\| rintracciarmi \|\| seguimi \|\| rintracciarci \|\| Problema di privacy \|\| Preoccupazione sulla privacy \|\| Preoccupazione per la posizione \|\| Rintracciarmi \|\| Seguimi \|\| Rintracciarci | datenschutzproblem \|\| datenschutz-bedenken \|\| standortbedenken \|\| verfolge mich \|\| verfolge mich \|\| verfolgen sie uns \|\| Datenschutzproblem \|\| Datenschutz-bedenken \|\| Standortbedenken \|\| Verfolge Mich \|\| Verfolge Mich \|\| Verfolgen Sie Uns | problème de confidentialité \|\| problème de confidentialité \|\| souci de localisation \|\| me suivre \|\| trouvez moi \|\| nous suivre \|\| Problème De Confidentialité \|\| Problème De Confidentialité \|\| Souci De Localisation \|\| Me Suivre \|\| Trouvez Moi \|\| Nous Suivre |
| inutili \|\| sciocchezze \|\| spazzatura \|\| Inutili \|\| Sciocchezze \|\| Spazzatura | nutzlos \|\| müll \|\| müll \|\| Nutzlos \|\| Müll \|\| Müll | inutile \|\| ordures \|\| des ordures \|\| Inutile \|\| Ordures \|\| Des Ordures |
| insetto \|\| passeggino \|\| Insetto \|\| Passeggino | fehler \|\| Fehler | punaise \|\| petit chariot \|\| Punaise \|\| Petit Chariot |
| impossibile installare \|\| non si installa \|\| impossibile installare \|\| Impossibile installare \|\| Non si installa \|\| Impossibile installare | kann nicht installiert werden \|\| wird nicht installiert \|\| konnte nicht installiert werden \|\| Kann Nicht Installiert Werden \|\| Wird Nicht Installiert \|\| Konnte Nicht Installiert Werden | ne peut pas installer \|\| n'installe pas \|\| impossible d'installer \|\| Ne Peut Pas Installer \|\| N'installe Pas \|\| Impossible D'installer |
| impossibile scaricare \|\| impossibile scaricare \|\| incompatibile \|\| Impossibile scaricare \|\| Impossibile scaricare \|\| Incompatibile | kann nicht herunterladen \|\| konnte nicht herunterladen \|\| unvereinbar \|\| Kann Nicht Herunterladen \|\| Konnte Nicht Herunterladen \|\| Unvereinbar | impossible de télécharger \|\| impossible de télécharger \|\| incompatible \|\| Impossible De Télécharger \|\| Impossible De Télécharger \|\| Incompatible |
| romanian | Spanish | czech |
| baterie \|\| scurgere \|\| Baterie \|\| Scurgere | batería \|\| desagüe \|\| Batería \|\| Desagüe | baterie \|\| kanalizace \|\| Baterie \|\| Kanalizace |
| spion \|\| spionat \|\| spyware \|\| Spion \|\| Spionat \|\| Spyware | espiar \|\| espiada \|\| espiado \|\| spyware \|\| Espiar \|\| Espiada \|\| Espiado \|\| Spyware | vyzvědač \|\| špehoval \|\| spyware \|\| Vyzvědač \|\| Špehoval \|\| Spyware |
| nu merge \|\| nu a funcționat \|\| nu funcționează \|\| Nu Merge \|\| Nu A Funcționat \|\| Nu Funcționează | no funciona \|\| no funcionó \|\| no funciona \|\| No Funciona \|\| No Funcionó \|\| No Funciona | nefunguje \|\| nefungovalo \|\| nejde to \|\| Nefunguje \|\| Nefungovalo \|\| Nejde To |
| prăbușire \|\| îngheţa \|\| Prăbușire \|\| Îngheţa | choque \|\| congelar \|\| Choque \|\| Congelar | pád \|\| zmrazit \|\| Pád \|\| Zmrazit |
| problema de confidențialitate \|\| preocupări de confidențialitate \|\| preocuparea locației \|\| urmărindu-mă \|\| urmărește-mă \|\| urmărindu-ne \|\| Problema De Confidențialitate \|\| Preocupări De Confidențialitate \|\| Preocuparea Locației \|\| Urmărindu-mă \|\| Urmărește-mă \|\| Urmărindu-ne | asuntos de privacidad \|\| preocupación de privacidad \|\| preocupación por la ubicación \|\| siguiéndome \|\| Sígueme \|\| rastreándonos \|\| Asuntos De Privacidad \|\| Preocupación De Privacidad \|\| Preocupación Por La Ubicación \|\| Siguiéndome \|\| Sígueme \|\| Rastreándonos | problém s ochranou soukromí \|\| soukromí \|\| obavy o lokalitu \|\| mě sleduje \|\| sleduj mě \|\| sledují nás \|\| Problém S Ochranou Soukromí \|\| Soukromí \|\| Obavy O Lokalitu \|\| Mě Sleduje \|\| Sleduj Mě \|\| Sledují Nás |
| inutil \|\| gunoi \|\| gunoi \|\| Inutil \|\| Gunoi \|\| Gunoi | inútil \|\| basura \|\| basura \|\| Inútil \|\| Basura \|\| Basura | zbytečný \|\| odpadky \|\| odpadky \|\| Zbytečný \|\| Odpadky \|\| Odpadky |
| gândac \|\| trăsură pentru două persoane \|\| Gândac \|\| Trăsură Pentru Două Persoane | insecto \|\| calesa \|\| Insecto \|\| Calesa | chyba \|\| buggy \|\| Chyba \|\| Buggy |
| nu pot instala \|\| nu se instalează \|\| nu a putut instala \|\| Nu Pot Instala \|\| Nu Se Instalează \|\| Nu A Putut Instala | no se puede instalar \|\| no instala \|\| no se pudo instalar \|\| No Se Puede Instalar \|\| No Instala \|\| No Se Pudo Instalar | nelze nainstalovat \|\| nenainstaluje se \|\| nelze nainstalovat \|\| Nelze Nainstalovat \|\| Nenainstaluje Se \|\| Nelze Nainstalovat |
| nu se poate descărca \|\| nu a putut descărca \|\| incompatibil \|\| Nu Se Poate Descărca \|\| Nu A Putut Descărca \|\| Incompatibil | no se puede descargar \|\| no se pudo descargar \|\| incomaptible \|\| No Se Puede Descargar \|\| No Se Pudo Descargar \|\| Incomaptible | nelze stáhnout \|\| nelze stáhnout \|\| nekompatibilní \|\| Nelze Stáhnout \|\| Nelze Stáhnout \|\| Nekompatibilní |
| portugese | Norwegian | lithuanian |
| bateria \|\| drenar \|\| Bateria \|\| Drenar | batteri \|\| tappe \|\| Batteri \|\| Tappe | baterija \|\| nusausinti \|\| Baterija \|\| Nusausinti |
| espiã \|\| espião \|\| espionada \|\| espionado \|\| spyware \|\| Espiã \|\| Espião \|\| Espionada \|\| Espionado \|\| Spyware | spion \|\| spionert \|\| spyware \|\| Spion \|\| Spionert \|\| Spyware | šnipas \|\| šnipinėjo \|\| šnipinėjimo programos \|\| Šnipas \|\| Šnipinėjo \|\| Šnipinėjimo Programos |
| não funciona \|\| não funcionou \|\| não está funcionando \|\| Não Funciona \|\| Não Funcionou \|\| Não Está Funcionando | fungerer ikke \|\| fungerte ikke \|\| jobber ikke \|\| Fungerer Ikke \|\| Fungerte Ikke \|\| Jobber Ikke | neveikia \|\| neveikė \|\| neveikia \|\| Neveikia \|\| Neveikė \|\| Neveikia |
| batida \|\| batido \|\| congelar \|\| Batida \|\| Batido \|\| Congelar | brak \|\| fryse \|\| Brak \|\| Fryse | avarija \|\| užšaldyti \|\| Avarija \|\| Užšaldyti |
| questão de privacidade \|\| preocupação com a privacidade \|\| preocupação com a localização \|\| me rastreando \|\| rastreie-me \|\| nos rastreando \|\| Questão De Privacidade \|\| Preocupação Com A Privacidade \|\| Preocupação Com A Localização \|\| Me Rastreando \|\| Rastreie-me \|\| Nos Rastreando | spørsmål om personvern \|\| bekymring for personvern \|\| beliggenhet bekymring \|\| spore meg \|\| spore meg \|\| spore oss \|\| Spørsmål Om Personvern \|\| Bekymring For Personvern \|\| Beliggenhet Bekymring \|\| Spore Meg \|\| Spore Meg \|\| Spore Oss | privatumo klausimas \|\| rūpestis dėl privatumo \|\| susirūpinimas dėl vietos \|\| sekdamas mane \|\| stebėk mane \|\| sekdamas mus \|\| Privatumo Klausimas \|\| Rūpestis Dėl Privatumo \|\| Susirūpinimas Dėl Vietos \|\| Sekdamas Mane \|\| Stebėk Mane \|\| Sekdamas Mus |
| sem utilidade \|\| lixo \|\| lixo \|\| Sem Utilidade \|\| Lixo \|\| Lixo | ubrukelig \|\| søppel \|\| søppel \|\| Ubrukelig \|\| Søppel \|\| Søppel | nenaudingas \|\| šiukšlės \|\| šiukšlių \|\| Nenaudingas \|\| Šiukšlės \|\| Šiukšlių |
| erro \|\| buggy \|\| Erro \|\| Buggy | bug \|\| buggy \|\| Bug \|\| Buggy | klaida \|\| pakvaišęs \|\| Klaida \|\| Pakvaišęs |
| não pode instalar \|\| não instala \|\| não foi possível instalar \|\| Não Pode Instalar \|\| Não Instala \|\| Não Foi Possível Instalar | kan ikke installere \|\| installerer ikke \|\| kunne ikke installere \|\| Kan Ikke Installere \|\| Installerer Ikke \|\| Kunne Ikke Installere | negaliu įdiegti \|\| neįdiegia \|\| nepavyko įdiegti \|\| Negaliu Įdiegti \|\| Neįdiegia \|\| Nepavyko Įdiegti |
| não pode baixar \|\| não foi possível baixar \|\| incompatível \|\| Não Pode Baixar \|\| Não Foi Possível Baixar \|\| Incompatível | kan ikke laste ned \|\| kunne ikke lastes ned \|\| uforenlig \|\| Kan Ikke Laste Ned \|\| Kunne Ikke Lastes Ned \|\| Uforenlig | negaliu atsisiųsti \|\| nepavyko atsisiųsti \|\| nesuderinamas \|\| Negaliu Atsisiųsti \|\| Nepavyko Atsisiųsti \|\| Nesuderinamas |
| danish | chinese | indonesian |
| batteri \|\| dræne \|\| Batteri \|\| Dræne | 排水\|\|电池\|\|排水\|\|电池 | tiriskan \|\| baterai \|\| Tiriskan \|\| Baterai |
| spion \|\| spioneret \|\| spyware \|\| Spion \|\| Spioneret \|\| Spyware | 间谍\|\|间谍\|\|间谍软件\|\|间谍\|\|间谍\|\|间谍软件 | mata-mata \|\| mata-mata \|\| spyware \|\| Mata-mata \|\| Mata-mata \|\| Spyware |
| fungerer ikke \|\| fungerede ikke \|\| virker ikke \|\| Fungerer Ikke \|\| Fungerede Ikke \|\| Virker Ikke | 不起作用\|\|不起作用\|\|不起作用\|\|不起作用\|\|不起作用\|\|不起作用 | tidak bekerja \|\| tidak bekerja \|\| tidak bekerja \|\| Tidak Bekerja \|\| Tidak Bekerja \|\| Tidak Bekerja |
| krak \|\| fryse \|\| Krak \|\| Fryse | 崩溃\|\|冻结\|\|崩溃\|\|冻结 | crash \|\| membekukan \|\| Crash \|\| Freeze |
| spørgsmål om beskyttelse af personlige oplysninger \|\| bekymring for beskyttelse af personlige oplysninger \|\| placering bekymring \|\| sporer mig \|\| spore mig \|\| spore os \|\| Spørgsmål Om Beskyttelse Af Personlige Oplysninger \|\| Bekymring For Beskyttelse Af Personlige Oplysninger \|\| Placering Bekymring \|\| Sporer Mig \|\| Spore Mig \|\| Spore Os | 隐私问题\|\|隐私问题\|\|位置问题\|\|跟踪我\|\|跟踪我\|\|跟踪我们\|\|隐私问题\|\|隐私问题\|\|位置问题\|\|跟踪我\|\|跟踪我\|\|跟踪我们 | masalah privasi \|\| masalah privasi \|\| masalah lokasi \|\| melacak saya \|\| melacak saya \|\| melacak kami \|\| Masalah Privasi \|\| Masalah Privasi \|\| Masalah Lokasi \|\| Melacak Saya \|\| Melacak Saya \|\| Melacak Kami |
| ubrugelig \|\| vrøvl \|\| affald \|\| Ubrugelig \|\| Vrøvl \|\| Affald | 无用的\|\|垃圾\|\|垃圾\|\|无用的\|\|垃圾\|\|垃圾 | tidak berguna \|\| sampah \|\| sampah \|\| Tidak Berguna \|\| Sampah \|\| Sampah |
| insekt \|\| buggy \|\| Insekt \|\| Buggy | 臭虫\|\|越野车\|\|臭虫\|\|越野车 | bug \|\| buggy \|\| Bug \|\| Buggy |
| kan ikke installeres \|\| installerer ikke \|\| kunne ikke installeres \|\| Kan Ikke Installeres \|\| Installerer Ikke \|\| Kunne Ikke Installeres | 无法安装\|\|未安装\|\|无法安装\|\|无法安装\|\|未安装\|\|无法安装 | tidak dapat menginstal \|\| tidak menginstal \|\| tidak bisa menginstal \|\| Tidak Bisa Menginstal \|\| Tidak Menginstal \|\| Tidak Bisa Menginstal |
| kan ikke downloade \|\| kunne ikke downloades \|\| uforenelig \|\| Kan Ikke Downloade \|\| Kunne Ikke Downloades \|\| Uforenelig | 无法下载\|\|无法下载\|\|不兼容\|\|无法下载\|\|无法下载\|\|不兼容 | tidak bisa mengunduh \|\| tidak bisa mengunduh \|\| tidak kompatibel \|\| Tidak Bisa Mengunduh \|\| Tidak Bisa Mengunduh \|\| Tidak Kompatibel |
| vietnamese | turkish | dutch |
| cống \|\| pin | boşaltma \|\| pil \|\| Boşaltma \|\| Pil | afvoer \|\| batterij \|\| Afvoer \|\| Batterij |
| gián điệp \|\| gián điệp \|\| phần mềm gián điệp | casus \|\| casusluk \|\| casus yazılım \|\| Casus \|\| Casusluk \|\| Casus Yazılım | spion \|\| bespioneerd \|\| spyware \|\| Spion \|\| Bespioneerd \|\| Spyware |
| không làm việc \|\| không làm việc \|\| không làm việc | çalışmıyor \|\| çalışmıyor \|\| çalışmıyor | werkt niet \|\| Werkte Niet |
| sụp đổ \|\| đóng băng | gürültüyle çarpmak \|\| dondurmak \|\| Dondurmak | crash \|\| bevriezen \|\| Crash \|\| Bevriezen |
| vấn đề riêng tư \|\| mối quan tâm riêng tư \|\| mối quan tâm vị trí \|\| theo dõi tôi \|\| theo dõi tôi \|\| theo dõi chúng tôi | gizlilik sorunu \|\| gizlilik endişesi \|\| konum kaygısı \|\| beni takip et \|\| beni takip et \|\| bizi takip et \|\| Gizlilik Sorunu \|\| Gizlilik Kaygısı \|\| Konum Kaygısı \|\| Beni Takip Et \|\| Beni takip et \|\| Bizi Takip Et | privacyprobleem \|\| privacyprobleem \|\| bezorgdheid over de locatie \|\| mij volgen \|\| mij volgen \|\| ons volgen \|\| Privacyprobleem \|\| Privacykwestie \|\| Bezorgdheid Over De Locatie \|\| Mij Volgen \|\| Mij Volgen \|\| Ons Volgen |
| vô dụng \|\| rác rưởi \|\| rác rưởi | yararsız \|\| çöp \|\| çöp \|\| işe yaramaz \|\| | nutteloos \|\| vuilnis \|\| vuilnis \|\| Nutteloos \|\| Vuilnis \|\| Vuilnis |
| lỗi \|\| lỗi | böcek \|\| buggy \|\| Hata \|\| Buggy | bug \|\| buggy \|\| Bug \|\| Buggy |
| có thể cài đặt \|\| cài đặt \|\| không thể cài đặt \|\| cài đặt | yüklenemiyor \|\| yüklenemiyor \|\| yüklenemiyor \|\| Yüklenemiyor \|\| Yüklenemiyor \|\| Yüklenemedi | kan niet installeren \|\| kan niet installeren \|\| kan niet installeren \|\| Kan Niet Installeren \|\| Kan Niet Installeren \|\| Kan Niet Installeren |
| có thể tải xuống \|\| tải về \|\| không thể tải xuống \|\| không tương thích | indiremiyorum \|\| indiremedi \|\| uyumsuz \|\| Indiremiyorum \|\| Indiremedi \|\| Uyumsuz | kan niet downloaden \|\| kan niet downloaden \|\| is niet compatibel \|\| Kan Niet Downloaden \|\| Kan Niet Downloaden \|\| Niet Compatibel |
| polish | malay | georgian |
| drenaż \|\| bateria \|\| Drenaż \|\| Bateria | longkang \|\| bateri \|\| Longkang \|\| Bateri | გადინება \|\| ბატარეა |
| szpieg \|\| szpiegowanie \|\| oprogramowanie szpiegowskie \|\| Szpieg \|\| Szpiegowanie \|\| Programowanie Szpiegowskie | pengintip \|\| pengintip \|\| perisian pengintip \|\| Perisik \|\| Pengintip \|\| Perisik | ჯაშუში \|\| ჯაშუში \|\| spyware |
| nie działa \|\| Nie Działa | tidak berfungsi \|\| Tidak Berfungsi | არ მუშაობს \|\| არ იმუშავებს \|\| არ მუშაობს |
| crash \|\| zamrażanie \|\| awaria \|\| Zamrażanie | crash \|\| beku \|\| Crash \|\| Freeze \|\| Beku | კრახი \|\| გაყინვა |
| problem dotyczący prywatności \|\| obawa o prywatność \|\| problem dotyczący lokalizacji \|\| śledzenie mnie \|\| śledzenie mnie \|\| śledzenie nas \|\| Problem Dotyczący Prywatności \|\| Obawa O Prywatność \|\| Problem Dotyczący Lokalizacji \|\| śledzenie Mnie \|\| śledzenie Mnie \|\| śledzenie Nas | masalah privasi \|\| kebimbangan privasi \|\| masalah lokasi \|\| menjejaki saya \|\| menjejaki saya \|\| menjejaki kami \|\| Masalah Privasi \|\| Kebimbangan Privasi \|\| Masalah Lokasi \|\| Menjejaki Saya \|\| Menjejaki Saya \|\| menjejaki Kami | კონფიდენციალურობის საკითხი \|\| კონფიდენციალურობის საკითხები \|\| ადგილმდებარეობის შეშფოთება \|\| თვალყურის დევნება \|\| თვალყურის დევნება \|\| ჩვენს თვალყურის დევნება |
| bezużyteczne \|\| śmieci \|\| śmieci \|\| Bezużyteczne \|\| Śmieci \|\| Śmiec | tidak berguna \|\| sampah \|\| Tidak Berguna \|\| Sampah | უსარგებლო \|\| ნაგავი \|\| ნაგავი |
| bug \|\| buggy \|\| Bug \|\| Buggy | pepijat \|\| buggy \|\| Bug \|\| Buggy \|\| Pepijat | bug \|\| buggy |
| nie można zainstalować \|\| nie można zainstalować \|\| nie można zainstalować \|\| Nie Można Zainstalować \|\| Nie Można Zainstalować \|\| Nie Można Zainstalować | tidak dapat memasang \|\| tidak memasang \|\| tidak dapat memasang \|\| Tidak Dapat Memasang \|\| Tidak Memasang \|\| Tidak Dapat Memasang | არ შეიძლება ინსტალაცია \|\| არ დააინსტალიროთ \|\| ვერ დააინსტალიროთ |
| nie można pobrać \|\| nie można pobrać \|\| niezgodne \|\| Nie Można Pobrać \|\| Nie Można Pobrać \|\| Niezgodne | tidak dapat memuat turun \|\| tidak dapat memuat turun \|\| tidak serasi \|\| Tidak Dapat Memuat Turun \|\| Tidak Dapat Memuat Turun \|\| Tidak Serasi | ვერ გადმოწერეთ \|\| ვერ გადმოწერეთ \|\| შეუთავსებელია |
| greek | arabic |  |
| αποστράγγιση \|\| μπαταρία | استنزاف \|\| بطارية |  |
| κατάσκοπος \|\| κατάσκοπος \|\| λογισμικό υποκλοπής | تجسس \|\| تجسس \|\| برامج التجسس |  |
| δεν λειτουργεί \|\| δεν λειτουργεί \|\| δεν λειτουργεί | لا يعمل \|\| لا يعمل \|\| لا يعمل |  |
| συντριβή \|\| πάγωμα | تحطم \|\| تجميد |  |
| ζήτημα απορρήτου \|\| ζήτημα απορρήτου \|\| ανησυχία τοποθεσίας \|\| παρακολούθηση \|\| παρακολούθηση \|\| παρακολούθηση | مشكلة الخصوصية \|\| مخاوف الخصوصية \|\| قلق الموقع \|\| تتبعني \|\| تتبعني \|\| تتبعنا |  |
| άχρηστα \|\| σκουπίδια \|\| σκουπίδια | عديمة الفائدة \|\| القمامة \|\| القمامة |  |
| σφάλμα \|\| λάθη | علة \|\| عربات التي تجرها الدواب |  |
| δεν μπορώ να εγκαταστήσω \|\| δεν εγκαθιστώ \|\| δεν μπορώ να εγκαταστήσω | لا يمكن التثبيت \|\| لا التثبيت \|\| لا يمكن التثبيت |  |
| δεν είναι δυνατή η λήψη \|\| δεν ήταν δυνατή η λήψη \|\| ασύμβατη | لا يمكن التنزيل \|\| لا يمكن التنزيل \|\| غير متوافق |  |

1. The privacy features have been extracted from official privacy policies published on the applications’ home page and Google Play Store page. [↑](#footnote-ref-1)
